# Enablers and barriers for hearing parents with deaf children: Experiences of parents and workers in Wales, UK

**DOI:** 10.1111/hex.13864

**Published:** 2023-09-11

**Authors:** Julia Terry

**Affiliations:** ^1^ Faculty of Medicine, Health and Life Science Swansea University Swansea Wales UK

**Keywords:** Bronfenbrenner, deaf, deafness, hard of hearing, hearing parents, support

## Abstract

**Background:**

More than 90% of deaf children are born to hearing families who know little about deafness. Benefits from hearing screening at birth are often lost, as families find little information about pathways for deaf children but are key to ensuring deaf children receive relevant language and communication support. Systems surrounding deaf children and family members are crucial for children's health and social development. Experiences of hearing parents raising deaf children and understanding factors that influence families' experience of navigating pathways for deaf children through health and education services are currently underreported.

**Methods:**

An exploratory study was conducted in Wales, UK. Twenty participants were interviewed, including 10 hearing parents of deaf children and 10 people who work with deaf children using semistructured interviews. Bronfenbrenner's ecological systems theory was used as a lens to explore the micro‐, meso‐, exo‐, macro‐ and chronosystems that surround children and families. This study explores potential supports and barriers in those systems.

**Findings:**

Findings are reported under two broad headings: enablers and barriers. Under enablers, it was found that provision of resources, supporting people and knowledge were key factors. Under barriers, a lack of knowledge, lack of provision and battling services and attitudes were key issues that need addressing.

**Conclusion:**

Hearing parents of deaf children in Wales, UK reported experiencing a range of enablers and barriers that impact upon their experiences of raising a deaf child. Further provision is needed by policymakers and governments to recognise support needs to improve the outcomes for deaf children.

**Patient or Public Contribution:**

This project was developed from initial discussions with the stakeholder reference group and progressed with the group's deaf panel and hearing parents with deaf children. The project's steering group was involved in study design, recruitment and continuous feedback on all stages of the research process.

## 1 INTRODUCTION

There are around 34 million deaf children globally.^1^ Evidence shows that 96% of deaf babies are born to hearing parents^2^, ^3^ who often have no experience of deafness and often do not know other people who are deaf, so it is vital that families benefit from a range of support processes and interventions in the early years of their child's life.^4^

Benefits from hearing screening at birth, when deafness is usually identified, are often lost, as parents of deaf babies may not be supported to develop vital skills for communicating with their growing children. Deaf children are at risk of not experiencing fully accessible language environments, which is a major factor that contributes to deaf population health disparities. Notably, deaf people are at greater risk of developing chronic diseases such as hypertension and diabetes in their lifetime.^5^

Families find little information or support available to navigate pathways for deaf children through health and education services, yet families are key to ensuring children who are deaf receive language and communication support. Communication challenges experienced by children who are deaf, and consequently their families, were difficult before the global coronavirus disease (Covid) pandemic, and much worse during, as not only were communications and interpersonal relationships made more difficult but also the lack of access to resources (information, education and health care), has increased vulnerability in child and adult deaf communities tremendously.^6^ Systems surrounding deaf children and family members are crucial in terms of children's health and social development, and subsequent achievements. Experiences of hearing parents raising children who are deaf, and identification of psychological and social factors that influence health and illness are currently under‐reported.

Hearing children are born into an accessible world with their language acquisition starting at birth and are usually able to master their native language by the time they are 5 years old.^7^ Deaf children's experiences are different because their access to language can be problematic with adverse consequences on their cognitive, social and emotional skills, as well as engagement in school, and academic outcomes. The most essential educational outcome for a deaf child is the mastery of at least one language, signed and/or spoken.^8^ In the midst of understanding about communication choices and language options for their deaf child are hearing parents who need support and guidance.^4^

Challenges are evident in navigating healthcare systems and the expectations of families and healthcare professionals, as well as the complex dynamics that exist between patients and providers.^9^ People who are deaf and/or their families can have a labyrinth of challenges to navigate regarding technological, interpersonal, linguistic as well as structural barriers, as they seek supportive and informative interventions.^10^ Whilst governments seek to improve services for people who are deaf or living with hearing loss, the increase in ageing populations means increasing need and challenge for all involved.^11^

Bronfenbrenner, a psychologist^12^ known for his ecological systems theory, identified that micro‐, meso‐, exo‐, macro‐ and chronosystems exist around a child and there are complex relationships across these systems that impact the child and their support systems. To understand the deaf child and their hearing parents' experiences, it is important to consider the interaction of environments that surround them.^12^ Bronfenbrenner's ecological systems theory was the chosen framework for this study because the influence of systems that potentially surround children and families plays a significant role in understanding supports and barriers of key health and development issues. Bronfenbrenner's dynamic framework is a lens to consider how individuals and their families engage and interact with different systems in their lives and communities (Table 1). Strengths of Bronfenbrenner's theory include the notion that individual differences between children and their families, as well as multiple influences, can all be considered, including the shared responsibility we have regarding child development and the creation of support systems needed to nurture development, as well as rich descriptions of the various environmental influences.^13^ Conversely, a limitation of Bronfenbrenner's framework is that it assumes systems are by and large supportive, and promote competence, which may not always be the case.^14^

**Table 1:** Bronfenbrenner's ecological system categories.

*Microsystems* refer to relationships and activities an individual engages with on a frequent or daily basis such as family, school, community, peers, faith settings and work.
*Mesosystems* are the interactions between the microsystems that a person experiences on a regular basis, for example, how often parents might engage with schoolteachers.
*Exosystems* are external to an individual that influence their microsystems and over which the individual has limited control and can include government, media, extended family and institutions.
*Macrosystems* are about greater cultural and societal structures that can influence the microsystem, mesosystem and exosystem.
*Chronosystem* is the overall system that represents the sociohistorical context and environmental events and transitions that occur for the individual, and within which the various systems operate.

It is logical that hearing parents with deaf children require support and systems in health, education and communities that will assist them in raising their children, but knowing about and navigating those intended supports and networks may be challenging and include both positive experiences and barriers and frustrations. Despite decades of research about children who are deaf being born to hearing parents, and methods that increase deaf children's language skills, development and learning, there is still limited research about knowledge and lived experience of navigating complex systems in health, care and education for families with children who are deaf. This study's aim was to establish insights into what supports hearing parents to raise a child who is deaf.

## 2 METHODS

This was a qualitative exploratory study using in‐depth individual interviews with hearing parents of deaf children, and people who work with children who are deaf, under 13 years of age and live in Wales, UK. The present study was carried out in compliance with the Consolidated Criteria for Reporting Qualitative Studies (COREQ)^15^ (see Table 2).

**Table 2:** Report on the accordance with the COREQ checklist for reporting qualitative research.

	Description
*Domain 1: Research team and reflexivity*	
1. Interviewer/facilitator	J. T. conducted all the interviews
2. Credentials	J. T. was the postdoctoral fellow on this project
3. Occupation	J. T. works as an Associate professor in the School of Health and Social Care at the Faculty of Medicine Health and Life Science, Swansea University
4. Gender	J. T. is female
5. Experience and training	J. T. has experience in qualitative research projects, including her PhD was a qualitative study on lived experience and talk. She underwent formal PhD education in social research methods
Relationship with participants	
6. Relationships established	There was no relationship between the researcher and the 20 participants. Eighteen participants were met for the first time at the interview and two were met initially at Deaf club presentations as part of the recruitment strategy
7. Participant knowledge of the interviewer	The participants had information that the researcher was from Swansea University and that the research was part of a postdoctoral‐funded fellowship. The participant information sheet and explainer video included information that the aim of the study was to find out what supports hearing parents with deaf children in Wales. If participants asked, J. T. told more about her background as a researcher
8. Interviewer characteristics	The main interest of J. T. is improving healthcare policy and practice in deaf communities
*Domain 2: Study design*	
Theoretical framework	
9. Methodological orientation and Theory	Underlying research paradigm was Bronfenbrenner's ecological systems theory, which views child development as a complex system of relationships affected by multiple levels of the surrounding environment, from immediate family and school settings to broader settings
Participant selection	
10. Sampling	Purposive sampling was sought through networks in Wales, including social media, charities, Health Boards and Education. Explained in this study
11. Method of approach	In the sampling phase, emails/social media messages were sent directly to organisations who had agreed to participate, who then promoted the opportunity to potential parents and workers with a participant information sheet and link to an explainer video. Those interested contacted the researcher via email, and then were asked to complete the consent form and agreed to an online interview time
12. Sample size	In total, 20 interviews were conducted: 10 hearing parents and 10 individuals who work with deaf children in Wales who are under 13 years of age. The workers were three speech and language therapists, four from Audiology sector and three from Education/Third sector
13. Nonparticipation	Fifteen parent participants came forward showing interest and completing consent forms, but only 10 resulted in being interviewed. There was no further contact with the five parents who completed consent forms but were not interviewed as there were no further communications from them, despite email reminders they gave no reason for not continuing to the interview stage
14. Setting of data collection	All interviews were conducted online using Zoom or Microsoft Teams due to the pandemic. At the time of study design (December 2020), face‐to‐face interviews were not an option due to Covid‐19. As restrictions eased, there were no requests from participants for a face‐to‐face interview
15. Presence of nonparticipants	At the online interviews, only the participant was present, except for a few parents with very young children who were occasionally in the same room as the participant
16. Description of the sample	Participant characteristics are shown in Table 3
Data collection	
17. Interview guide	A topic list was used during the questions. Questions centred around journeys for deaf children and their families. Because of the semistructured nature of the interview, the topic list was used to give guidance to the interviews but was not binding for the content of the interviews. The topic list was adjusted throughout the interviewing phase of the research with prompts
18. Repeat interviews	No repeated interviews were carried out with the participants, as it was not required for the study
19. Audio/visual recordings	All online interviews were recorded with digital audio and visual (mp4) files downloaded and stored on the author's password‐protected computer and university server
20. Field notes	J. T. made field notes during and after each interview. Notes included observations, thoughts and information points. A reflective journal was also kept during the study. Both field notes and journals were used to inform the analysis of data
21. Duration	The duration of interviews varied between 47 min and 1 h 26 min
22. Data saturation	Data saturation was discussed in supervision; from the outset of study, the plan was to recruit 20 participants, which was achieved, aiming for a range of location, role, gender, age of deaf child and worker role
23. Transcripts returned	Transcripts were not returned to participants as talk is at a moment. To ensure faithfulness to recordings, transcripts were read several times for accuracy while listening to the recordings with necessary corrections made
*Domain 3: Analysis and findings*	
24. Number of data coders	The first author performed the open coding of the data. The steering group and supervisor participated in the discussion and naming of codes. Information on the coding of the data is provided in the Section 2 of this paper
25. Description of the coding tree	No coding tree was used. Themes were derived from the data using thematic analysis
26. Derivation of themes	The themes were derived from the data and were discussed and agreed on by the researcher, supervisor and steering group. Early codes and descriptors were discussed with the steering group and study supervisor, along with anonymized data extracts to minimise bias, confirm themes and to develop knowledge
27. Software	QSR NVivo 12 was used, and a list of free nodes was created and extracted into themes
28. Participant checking	As explained in 23, there was no initial feedback to participants on the findings
Reporting	
29. Quotations presented	The themes in Findings section are illustrated by participant quotations. Each quotation is identified by a participant number and pseudonym
30. Data and findings consistent	The presented data and findings are consistent
31. Clarity of major themes	Findings are presented under two major headings: enablers and barriers. The major themes are presented through the lens of Bronfenbrenner's ecological systems theory, which was used as a framework for the analysis
32. Clarity of the minor themes	Minor themes are evident in tables with details in Section 2 of this paper

Abbreviation: Covid‐19, coronavirus disease 2019.

### 2.1 Design

Qualitative approaches allow researchers to explore experiences and perspectives on what supports hearing parents with deaf children to improve structures that exist in health, social care, education and community settings.^16^ Bronfenbrenner's ecological systems theory^12^ was used as a lens to guide the development of the study. Parents and workers were interviewed one‐on‐one to create a safe space for them to share their experiences. The scoping literature review part of this project included a review of current evidence concerning the support systems and structures surrounding deaf children with hearing parents,^4^ with a protocol published on Open Science Framework (https://osf.io/w48gc/). The funding application was written during the pandemic, and the study commenced in April 2021. From the outset, all research activities were online, with acknowledgement that data include reference to experiences before the pandemic, during, and then later as Covid‐19 restrictions began to ease.

### 2.2 Study setting and participants

Whilst the intended participant group were hearing parents with deaf children in Wales, equally hearing grandparents, hearing kinship parents or hearing foster parents were considered if they had been regularly involved with the child's care since birth. The other participant group was people who work with children who are deaf in Wales under 13 years of age, who were from education, health and deaf charities. All participants were over 18 years of age, and a range of participants was sought to reflect diversity incorporating a mix of genders, people with children of different ages and from different parts of Wales. The actual settings for data collection were through video conferencing platforms, for example, either Zoom or Microsoft Teams, using semistructured interviews.

Parent participants included nine females and one male. Worker participants included eight females and two male workers. Participants were recruited via social media (*n* = 5), local Health Boards (*n* = 7) and third‐sector, education, local authority and word of mouth (*n* = 8). Workers were from Audiology, Education, Third sector and Speech and Language Therapy (Table 3 provides participant details). The total amount of recorded interview data was 1331 min. Geographical areas of Wales and the gender of deaf children are not displayed in the participant characteristics table to protect participant identity.

**Table 3:** Participant characteristics.

	Parent pseudonym	Age of deaf child (in bold)	Time
1.	Anna	**7**	1 h 9 min
2.	Bella	**11**	1 h 5 min
3.	Charlotte	**11 months**	55 min
4.	Diane	Older sibling, **6**	49 min
5.	Eunice	Older sibling, **3**	1 h 8 min
6.	Flora	**17 months**	1 h 3 min
7.	George	**9**	1 h 15 min
8.	Hayley	**4, 2**	1 h 3 min
9.	Ingrid	5, ***7***, 8	1 h 21 min
10.	Jade	13, 12, **9**, 7	56 min

	Worker pseudonym	Work area	Time
1.	Kayleigh	Speech and Language Therapy	1 h 13 min
2.	Lisa	Speech and Language Therapy	1 h 26 min
3.	Mark	Audiology	1 h 3 min
4.	Nina	Education	1 h 13 min
5.	Olivia	Audiology	54 min
6.	Penny	Audiology	47 min
7.	Ruth	Third sector	1 h 10 min
8.	Sarah	Speech and Language Therapy	1 h 11 min
9.	Tanya	Education/Third sector	1 h 19 min
10.	Vic	Audiology	1 h 11 min

### 2.3 Recruitment

Parent/family participants were sought through deaf charity stakeholder groups including the National Deaf Children's Society, Deaf Child & Young People Youth Clubs and through deaf social media groups including Facebook pages (all were Wales, UK‐based). Recruitment was also sought through deaf units in schools in Wales. Parent and worker participants were also sought through the National Health Service by teams and individuals mentioning the opportunity to take part. Purposive sampling was carried out to maximise the diversity of participants from different backgrounds and settings and geographical regions of Wales, through deaf charities, social media, and health, care and public sectors.^17^

Potential participants were sent a flyer (circulated via stakeholder groups/on social media), which included a link to an explainer video on YouTube (https://youtu.be/f8MYDcKqhsI). The study information was available on a web platform, where the landing page supplied a link to the short explainer video about the opportunity to participate in the study (above). The web platform also housed the relevant participant information sheets for parents and workers, respectively, including easy‐to‐read versions, as well as a link to the study's consent form. All study materials were approved by the SUPERSTAR project steering group. Participants were also able to express an interest by emailing the researcher directly and receiving information by email as attachments. The sample size of this study was 10 hearing parents of deaf children under 13 years of age, and 10 workers. For this study, a total of 20 participants was predetermined at the funding application stage as feasible for the project aim and timeframe.

Invitations to participate were sent out periodically from October 2021 to October 2022 through social media, and local collaborators approved through ethics, which included local Health Boards in Wales, UK. During the recruitment phase, responses from potential participants were slow. All 10 workers who volunteered to participate were interviewed. Fifteen parent participants came forward showing interest and completing consent forms, but only 10 resulted in being interviewed. There was no further contact with the five parents who completed consent forms but were not interviewed as there were no further communications from them, despite email reminders they gave no reason for not continuing to the interview stage.

### 2.4 Interviews

Interviews provided the opportunity for parents and workers to report their experiences about supports, networks and information for hearing parents of deaf children in Wales, UK. Interviews were undertaken with workers in education, health, care and third‐sector settings, with relevant permissions sought, to gain multiple perspectives. Consent to record and store data was obtained at the outset with interviews anonymized and identifying features removed to ensure anonymity, and any identifying information stored separately from interview material. A reflective journal, as well as field notes for consideration, were used postinterview, and throughout the project. Field notes were kept capturing relevant thoughts, concepts and background context.

Interviews were conducted between November 2021 and December 2022 over Zoom or Microsoft Teams and lasted approximately 50–80 min. Each participant recounted their experiences extensively, with no ill‐effects apparent, and all appeared positive about the interview experience. The interview schedule is available as an additional file and includes open‐ended questions. After starting with a brief geographical question about where participants live/work, parents were asked ‘tell me about when you first realized your child was deaf’ and for workers interviews started with ‘tell me about your role working with deaf children and their hearing parents in Wales’. Each interview concluded with a short debrief as well as further support information sent by email postinterview (which included information about primary healthcare, mental health support lines and deaf charities).

### 2.5 Data analysis

All the interviews were digitally recorded with transcript files saved and prepared for analysis by listening to each interview and correcting transcripts, so they appeared verbatim. Transcripts were read and reread using a process of familiarisation, identifying potential themes, charting and mapping.^18^ First, initial codes were generated and collated manually using a summary page for each interview transcript, which was data‐driven, and reduced large amounts of data from each transcript into a more manageable form.^19^ Second, QSR NVivo 12 was used, and a list of free nodes was created and extracted into early themes. Early subthemes and descriptors were discussed with the steering group and study supervisor, along with anonymized data extracts to minimise bias, confirm themes and to develop knowledge (see Table 4).

**Table 4:** Early consideration of subthemes using Bronfenbrenner ecological systems as a lens.

	Enablers	Barriers
Chrono	Models Pathways	Time
Macro	Language access	Impact of bureaucracy Attitudes
Exo	Deaf club Deaf friendly Systems and communities	Lack of deaf CAMHS Navigating services British Sign Language classes—cost British Sign Language classes— lack of
Meso	Connect with other parents—individually Connect with other parents—group Connect through resources	Not knowing Lack of staff knowledge Not connecting with other parents
Micro	Family Teacher of deaf CYP Information Other parents Emotional support	Expectations of parents before birth Lack of access
Individual	Inner strength	All‐consuming

Abbreviations: CAMHS, Child & Adolescent Mental Health Services; CYP, children and young people.

During the analysis process, Bronfenbrenner's ecological systems theory was used as a lens to guide interpretation. Framing problems within a socioecological systems framework uncovers their complexity and can show underlying sociocultural factors and points of power.^20^

Data memos and visual notes were kept during the analysis process, and a reflective journal assisted reflexive processes as decisions around theming were considered. Aware of potential researcher bias and that deaf networks are considered small‐connected communities,^21^ particularly in a small country like Wales, the steering group was a useful intermediary and provided professional feedback on progress. Each stage of data analysis was also discussed in supervision with the project supervisor providing oversight.

### 2.6 Public and patient involvement

The research idea originated during two public engagement events during 2020, the research proposal developed further in discussion with the deaf panel of our Deaf mental health research network, and then a project steering group was established with an independent Chair, lay members, deaf people, parents of deaf children and stakeholders who work in health, care, education and deaf charities. After terms of reference for the project steering group were agreed upon, the group members were involved in shaping the research throughout. For example, discussing key areas and questions for the qualitative interviews with hearing parents and people who work with children who are deaf. The group was an expert advisory group and was not a gatekeeper. The advisory group recommended that easy‐read documentation be available throughout this project, hence easy‐read versions of the participant information sheets and consent form were as inclusive as possible. The group informed and influenced the preparation of interview schedule, data analysis, preparation of findings and presentation for dissemination.

## 3 FINDINGS

**Figure 1:**
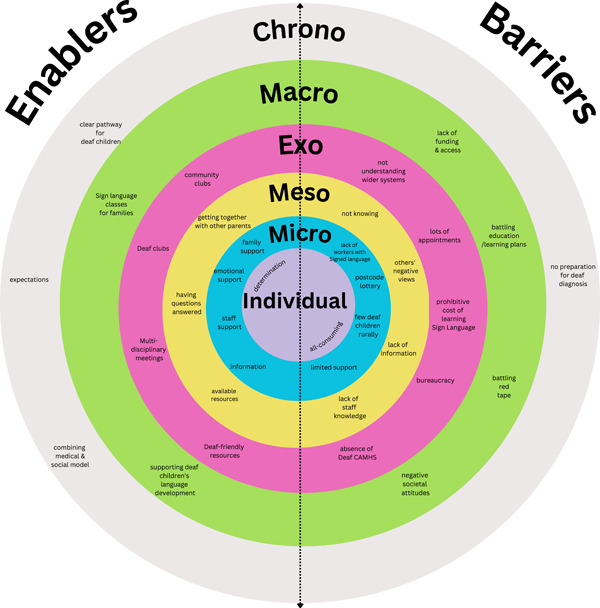
Bronfenbrenner ecological systems for hearing parents with children who are deaf.

Overall, experiences are reported in this section under two main headings: enablers and barriers, with each of Bronfenbrenner's systems used as a subheading and then the majority of subthemes reported in named subsections (see Figure 1).

### 3.1 Enablers that support hearing parents with children who are deaf

Participants spoke about what enabled and supported parents from their observations and experiences.

#### 3.1.1 Individual

The individual, according to Bronfenbrenner, is the child/family and the systems reported below are those that surround them.

##### Determination

Parents spoke about their own attributes, temperament and characteristics, and their awareness of how helpful their own individual motivation was an enabler in their view.

I know that if you push hard enough you get it. I am like a dog with a bone, and that's my personality. (Hayley, P8)

#### 3.1.2 Micro

##### Family support

Microsystems refer to the people that hearing parents regularly encounter, such as other family members, teachers and health workers.

my mum's been amazing … she's come to all appointments with me, and … because obviously you don't … remember to say this, this and this all the time. You do forget a little bit. So it's been nice to have her as a little ‘you've got to say that now’. (Jade, P10)

##### Staff support

Parents often spoke about teachers of deaf children and young people who had been assigned to them, as often this was the person co‐ordinating their child's care and support.

our teacher the deaf came out … within the first couple of weeks, and she was amazing because she just spoke to us about … the normality of deafness and what life would look like, what education would look like. She was really encouraging, … to do with the support in the county, and so I think, where we live has got a huge amount of support for deaf children and that's what we've experienced. And I feel that people have gone above and beyond, to accommodate what we wanted as a family. (Eunice, P5)

##### Information

Parents reported that support came in the form of information from workers:

They like to know what are the consequences later on what the children may need in the future to prepare them—if it's temporary loss they like to know the time scale … or when the surgery happen or … to know everything what will happen and that's empowering them as well. (Olivia, W5)

##### Emotional support

Also, emotional support was so important:

I had to ring my husband and say he's failed the hearing test … Then I rang my mum. Wailed down the phone to my mum. And then this amazing [member of staff] who I will also never forget, just like battered the door open, pushed this new born hearing screening lady out the way who was standing there going ‘oh I'm really sorry’, and just scooped me up in her arms, and just literally hugged me and didn't let me go for about ten minutes. And she was like ‘You're all right. I got you, love, you're all right’, and … to this day I will never forget her. Really important. (Hayley, P8)

#### 3.1.3 Meso

Mesosystems are the connections and communications that exist between microsystems. Examples from participants included connecting with other parents, and how they engaged with groups of other hearing parents with deaf children on a social level and in terms of emotional connections.

##### Getting together with other parents

we … had a few get togethers in a park where just lots of other mums would come and we'd sit … on a blanket and … bring musical instruments, and we do some sing songs with sign … it was just a chance to meet other parents really and then we've got a WhatsApp group just for the parents. (Charlotte, P3)

I go to the parenting club and that really helps me because I was so scared from the surgery … and there was some parents that helped me go through that, they just give me their experience like they told me … what's happening with their children, and I can be in touch with them, I can see their children speaking and that was a big relief for me because I was scared. (Flora, P6)

Parents spoke about how parent groups were sometimes arranged, or they organised these independently.

… the teachers of the deaf have done several family days, which has been brilliant … there's a couple of mums that have got a [child] around [own child's] age that I make an effort to meet up with for coffee. Because obviously I want [own child] to have deaf friends. (Eunice, P5)

Parents noted while they connected with other hearing parents, they saw value in their children interacting too:

the parents could get together and chat and the kids could go and be deaf kids with all the deaf kids and not feel different. (Bella, P2)

Sometimes parents noted that workers would attempt to facilitate connections with other parents individually.

The schoolteacher actually invited us in to have an informal chat with [another child's] parents, because she knew that we will be starting to discuss where [our child] would go for comprehensive school in two years' time. So she thought it would be useful for us to have a chat together. (George, P7)

##### Having questions answered

In terms of information and resources being enabling, a participant from Audiology noted that parents have the opportunity for:

quite an open dialogue where they have chance to not only digest what we've told them, but … time to have questions answered. We also support with take home leaflets, because in these appointments … a lot of information is discussed. A lot of it may be … a bit of a shock. So it's really important that they have information that they can read in their own time, but they will also always get a copy of any clinical letters. And we'll make it clear that we are available for contact, if they do have any additional questions, because it's quite easy to attend an appointment and things that you had the intention is to ask, go out your head. (Mark, W3)

##### Available resources

Workers noted they needed to change ways of working during the pandemic and many spoke about making online resources and videos. Nina said:

we've managed to have funding to buy books for parents and … post out to the parents to be able to use and so there's been a lot more accessibility of things that parents can access without needing us to be there. (Nina, W4)

There was a sense of equipping parents more with tangible resources and guidance, rather than overrelying on interventions from workers themselves.

#### 3.1.4 Exo

Exosystems are wider systems and contexts that impact on individuals such as wider groups and government, as well as systems and communities.

##### Community clubs

When he was very young, I took him to like a little football club thing, … and they were very happy to give him the extra help that he was gonna need, you know. (Jade, P10)

##### Deaf clubs

They also talked about the value of engaging with Deaf clubs:

for hearing families, you know, I try and talk to them about [Deaf clubs] all this out there that you can access, you know, which might be a really good support network for you but also for you to start to understand a bit more about deafness and Deaf culture and the challenges your child may face there, without that exposure to Deaf community, or Deaf culture, you just wouldn't know or understand. (Lisa, W2)

And how Deaf clubs can offer something unique for deaf children themselves. One‐third‐sector charity spoke about a group they run for young deaf people, and how parents were aware of the positive changes this could bring about in their children, who had previously been withdrawn about their deafness:

by week three it's both hearing aids on, walking loud, tall, and proud of Deaf identity, because he was surrounded by other children wearing hearing aids, using BSL or directing one of the others to use BSL, complete change, massive increase in his confidence. If we get the transitioning correct from [childrens club], into the youth club, into volunteering, into employment. (Ruth, W7)

##### Multidisciplinary meetings

The experiences of enablers during the pandemic arose in conversation and depended on the parents' or workers' experiences of support pre‐ and postpandemic as they gave examples and made comparisons. Many parents and workers spoke about being engaged with many members of the multidisciplinary team on finding out their child was deaf. Whilst these meetings had occurred pre‐Covid‐19, having these meetings online was beneficial for some.

we've had like … multidisciplinary team meetings, we've had therapists—speech and language, teacher of the deaf in on the same kind of session. To see how [child's] doing so that they're all on the same page so that I think has been amazing that would have never happened without the technology of Zoom. (Eunice, P5)

##### Deaf‐friendly resources

Parents also talked about the help they found from deaf‐friendly resources that they had learned about from connecting with others in wider systems, such as over social media. Examples in people's talk came up too in relation to aids and devices.

A lot of the parents, they like a lot of support around technology …environmental aids, flashing lights. Alarm clock, those are the sorts of things that they want more support with. (Nina, W4)

Parents often spoke about online communities where they either posted questions or simply read posts and found the information enabling and beneficial.

Things like sport and hearing aids so I always say to my husband ‘Oh, I found this’ … and it generally it is helpful … like Christmas presents and … books that have characters that have hearing aids or dolls that have hearing aids like I wouldn't have thought about those things if I wasn't part of that [online] group. (Charlotte, P3)

#### 3.1.5 Macro

Macrosystems are broader contexts that include cultural, socioeconomic and political elements of systems. Examples of enablers in this subtheme include participants' talk about access to language support, particularly in terms of learning British Sign Language.

##### British Sign Language classes for families

Parents showed awareness of the importance of British Sign Language.

even if they're … if they are deaf, they don't have a problem, they have BSL and most of the … parents that have their children, they need to give them that opportunity, just to have a language. (Flora, P6)

##### Supporting deaf children's language development

Parent participants also were aware that other minority languages have more recognition and accompanying resources because of their legal status. The Welsh Language Act 1993 established that in the course of public business and the administration of justice, so far as is reasonably practicable, Welsh and English should be treated equally. The BSL act in England, UK means legal recognition, although there is limited provision for parents to learn British Sign Language easily.

…when they do ask me for advice [I say] ‘you need to learn BSL’. And it's ‘whoa that's a bit hard that is’. The families who do learn BSL, I'd say 90% it's the mother who learns it, of course it's the cost involved too. If I looked on the internet I could probably find an introductory Welsh class for free. Because the Government will fund it. But there are children out there who can't even communicate with their mothers and fathers. (Tanya, W9)

I would like British Sign Language used in the early years with him, and you know, I was told … Well, you don't use British Sign Language at home … And then, my answer was well if I didn't use Welsh at home, but I wanted [child] in a Welsh nursery school I'm sure I'd get all the help in the world. (Anna, P1)

Parents were aware that British Sign Language classes could be enabling if they were provided. A few had managed to source some, but were aware of the political and economic elements of provision.

#### 3.1.6 Chrono

Chronosystems are human ecology systems that continue to change over time. Participants mentioned there is much potential for the development of clear pathways for deaf children through health and education services and that this would help parents and workers to know routes and what to expect.

##### Clear pathway for deaf children and expectations

Health boards [should have a] definite pathway … means that parents will have a clearer route through the service and that when they talk to each other, they'll be able to share experiences and say ‘oh yes … we've had that and that's what you do first’, so a lot of it is around training and making sure parents feel empowered … so it's following a pathway, rather than just we think this is going to work, we know this works, because it's evidence based. It helps set up expectations from [the start] which I think for parents the aim of a pathway is to make services equitable, they need to know what's next, what to expect. (Kayleigh, W1)

##### Combining medical and social model

Both parent and worker participants showed an understanding of the different models of deafness, which identify how deaf people can be viewed and treated. For example, the medical model of deafness is about enabling hearing, providing aids, and providing a ‘cure’ to what can be seen as a problem.^22^ In contrast, the social model of deafness sees it as a disability with environments that can enable deaf people by providing access and making reasonable adjustments.^23^ Conversely, the deaf cultural model values deaf people as a cultural and linguistic group who use a signed language and are proud of their deaf heritage.^24^ Participants recognised that different model and service approaches had an impact on people's experiences.

From a medical model I think it's brilliant but from a social model that, you know, there's a lot needs to be done, but that's not the job, the job is the medical model, so there needs to be something in place …: almost like what the third sector is … because the medical model is fantastic, but the social and the medical model should be one. (Anna, P1)

### 3.2 Barriers

Barriers as reported by participants about support for hearing parents with deaf children, using a lens of Bronfenbrenner's systems.

#### 3.2.1 Individual

##### All‐consuming

The determination to get support was apparent, but parents showed awareness that their own responses might be unhealthy and pose further difficulties.

My experience is that, you can either switch off and step back because it's too much to think about, too hard to process or you can go the opposite, like me, and get obsessed, no place is healthy! (Anna, P1)

#### 3.2.2 Micro

Parents and workers spoke about barriers as they saw them in microlevel systems and how these reduced or made supporting parents difficult.

##### Few deaf children rurally

Participants spoke about geographical areas where they lived and worked and were aware that, particularly in rural areas, support and opportunities were limited.

The main challenges of parents, I think, is the access to support group I think … that's what we lack here … and it's just a difficult one. (Penny, W6)

##### Lack of workers with British Sign Language

Parents noticed times when there were simply no workers with the required skill or knowledge set to support their deaf children.

I contacted all the nurseries which obviously take babies in the area. There's no one in any of the nurseries who can do any BSL. (Ingrid, P9)

[it's] uber difficult, like he was lucky there for a few years that he had that one that could do British Sign Language. But the rest you know, people don't even know, apply for the job when they advertise it, because nobody's got that skill in the area, you know. (Jade, P10)

##### Limited support

Parents expressed they felt support was limited with many reporting feeling isolated and that they were journeying alone, even when some elements of support had been positive.

I think as parents we've largely been left to it. We've muddled our way through. I think [child] has been quite well supported, but for us as parents again, I'm not quite sure what I want either I just don't feel that there has been much support for [spouse] and myself. (George, P7)

#### 3.2.3 Meso

##### Not knowing

As parents discovered their child was deaf, they began to engage with services and reported their experiences and their views on the information provided by services. Parents often felt they simply ‘did not know’ or ‘did not know what to think’ of the information offered.

I don't remember there being kind of any practical things that they offered, I do remember from the outset being told ‘Do not sign, do not learn to sign with your child because [child] can hear now with hearing aids and if you sign, … and [child] becomes reliant on sign, they will never learn to talk’. (Bella, P2)

All parents that I work with love their children, but they don't know how to act as advocates for them because they don't know what to ask for. (Kayleigh, W1)

##### Others' negative views

Workers spoke of families who had struggled with care decisions due to attitudes and views from other family members who had strong negative views but were not in contact with services and came from uninformed positions.

without … knowledge … they were telling the parents of this [child] … that the operation's risky, too risky, and that they shouldn't do it … they did eventually go through the procedure. And they are so glad that they've gone through that process because [child] is just making so much progress now. And they said to me they wished they never listened to the elder family members or the community members because … those people put the fear in the parents. (Vic, W10)

##### Lack of information

Parents reported feeling out of the loop when they were not informed of changes to their child's care, education or support.

they don't keep me informed what's going like I didn't even know they'd changed the Teacher of the Deaf, which I think is out of order. (Diane, P4)

Whilst some workers voiced the potential benefit of providing telephone numbers of other parents with deaf children (with permission) for support, several parents voiced negative experiences of this, and did not feel it was appropriate.

I was given two numbers. Two mothers to speak to at the time and obviously I was very desperate to speak to them. But in hindsight, you know those mothers… bit busy… it's not really their role … and I couldn't actually get in touch with them. Very isolating time extremely isolating … those mothers, they're not trained to give me what I needed. [and at a later point] I was given the number of a mum who wasn't the right person to me to meet. (Anna, P1)

##### Lack of staff knowledge

Parents frequently spoke about staff lacking knowledge about children who were deaf.

the health visitor was asking us about … how we was going? How was our experience with him, she didn't have any ideas of where to direct us or anything. (Ingrid, P9)

a bit kind of outside of what they normally deal with …, she said, ‘oh no I've never had a [deaf] baby before’ and so she couldn't answer my question or couldn't get back to me … It made me like panic … the question was quite a basic question … so I think I just quickly learned that that's not where I went to for … more specific help. …Obviously I'm a bit sensitive but it felt a little bit like people were a bit embarrassed about [child's] hearing aids like when they come to the point when … they wanted to weigh [child] … like it was this big elephant in the room … I just felt like they were very much an issue. (Charlotte, P3)

To these parents there were plainly barriers they spoke of in relation to staff's attitudes and knowledge about their deaf child's care and treatment, which did little to encourage or support them as hearing parents.

#### 3.2.4 Exo

##### Not understanding wider systems

Both parents and workers reported that not knowing how wider systems worked or how to access help and support was an ongoing issue. In some respects it was the workers from Deaf charities who spoke about this vociferously; they said that this was because they were outside statutory services and had a wider lens.

[I spend a lot of time] pointing them in the right direction of who they need to speak to. Because they just get so many barriers Schools don't know who to put them in touch with either. People not knowing is a big part of the problem. Who do I go to? Where? Well I need that person to get that information, but I need to go there to get [something else]. There is no one‐stop shop unfortunately. (Tanya, W9)

##### Prohibitive cost of learning British Sign Language

Participants spoke about the frustrations and barriers they had encountered if they had tried to access learning British Sign Language.

If you have a child who has any form of a specific need then your time your energy will be spent a lot … back and forth to doctors appointments [and] everything else, and the fact that it's not readily available for parents to learn BSL And if they want to learn, then it's a it's a high cost, especially if there are other family members … that want to learn as well. (Ruth, W7)

I try to learn British Sign Language but this is difficult, you know. And people say there's not a lot of support out there for families to actually do that in the first place. But to get funding. But then Covid kicked in, and kind of that was the end of that, you know. But yeah, you know, I can't afford £800, whatever it is to put myself on it again, you know, [I] just use the apps and teach myself that way. (Jade, P10)

##### Bureaucracy

Parent participants voiced concerns about the lack of clear pathways, as well it feeling that their deaf children had to fit into bureaucratic systems rather than provision being accessible and available.

There isn't really a protocol or a way things are done or a pathway for anyone to follow necessarily. And so it took it took me and my husband throwing our toys out the pram a little bit [to get help]. (Eunice, P5)

I think, unfortunately, conversations that I've had with lots of hearing parents is that their children aren't deaf enough. And so I think a lot of children, especially preschool children, will have hearing aids, and that's it. They'll probably have a six monthly check up with audiology, and parents are left to kind of just get on with it. (Hayley, P8)

#### 3.2.5 Absence of deaf CAMHS

Workers particularly were aware of services that were currently absent in Wales.

In terms of having a specific deafness CAMHS, we don't currently have access to that in Wales, whereas in England, they do. So there are some gaps in terms of services. (Lisa, W2)

I don't know where the nearest Deaf CAMHS is for Wales. Well, we should have it because we know that there's a much higher risk of mental health difficulties and there are language barriers for these children. Yeah, you know it's one of those things it's like, if we can get it and push for it. You know we want it, but it's a service that they can't possibly access. (Sarah, W8)

#### 3.2.6 Macro

##### Lack of funding and access

Parents showed awareness of wider systems that they and their children were subject to and that this had implications for what might or might not be provided.

We still didn't know what he was really entitled to because everybody that we spoke to … it came down to funding and so everybody was very quickly kind of passing the buck. (Bella, P2)

##### Battling education/learning plans

In terms of education support, having a statement of educational needs/individual learning needs plan came up frequently.

If you've got a statement, you've got in black and white that [child's] entitled to one [without it] you've got nothing to stand on … everyone had to fill up the form and send it to the Council about … [child's] needs. But one piece of evidence was missed … sent back late … there's a six month timeframe … I [phoned] the Council … I was told that because a piece of evidence had gone in late … it would take longer … I think it took us a year to get this statement done. (Ingrid, P9)

##### Battling red tape

Parent participants spoke of the barriers within systems that impacted staff and how they were

being cynical to the system … I am pretty convinced it's just the fact that [child's] just too quiet for them to notice … And that worries me … [child is] now on his fourth teacher of the deaf, because they don't stay … our funding here is always a temporary funding through to the rest of following year, and when they then say to our Teachers of the Deaf ‘we don't know yet if we can fund your contract next term … because we have fixed rate funding’, and so they leave. (Bella, P2).

Workers also were aware of the complexities and rigidity of red tape as a barrier to parent support.

there are processes and procedures that have to be in place, but sometimes … the processes and procedures become too rigid they stop being just a framework for you to work within and they become a stick with nails in the end, [that] beat everybody over the head … And when you have fundamental needs, … you need flexibility, you need that ability to be warm to be welcoming to present something different, an opportunity for people. (Ruth, W7)

##### Negative societal attitudes

Parents themselves were aware of wider societal attitudes towards deaf people and how this impacted on them and their children.

so one thing that does happen in our life all the time is horrible, [it's] people coming up to us … and commenting on [child's] hearing aids … making these comments that have to do with shame and it happens … hundreds of times. And that's like a really difficult thing, especially when you've got an older child that's going to absorb those comments that was something shameful about the situation … something I need to learn … is how to respond in a way that's helpful … for my children … not confrontational. I think that's one of the hardest things of our whole experience is just people … and … some of my friends say … ‘why don't we take them [hearing aids] out?’ because we don't want to get comments … so like a family wedding, I just take them out so we've not got 100 people asking. (Charlotte, P3)

#### 3.2.7 Chrono

##### No preparation for deaf diagnosis

Participants spoke of their early feelings on finding out their child was deaf.

Finding out [child] was deaf quite early on, I think was a bit of a shock. We never met anyone who was deaf, so we'd never been around [anyone] who was deaf … Nothing, no BSL, no deaf people, nor anything so obviously … it was a bit of a shock, and it was a bit like what we do now? (Ingrid, P9)

Whilst their knowledge and feelings may have changed over time, there was an awareness that there was no preparation, that parents expect a healthy baby and that perhaps more awareness in health staff, such as midwives and other health staff could help prepare parents more about the potential for having a deaf child, and how a diagnosis of deafness is delivered in practice.

## 4 DISCUSSION

### 4.1 Discussion of findings

This study aimed to explore the support and structures for hearing parents with children who are deaf, under 13 years of age within Wales, UK and reported a range of enablers and barriers mentioned by parents and workers. Findings corroborate those of previous studies where families were keen to provide nurturing environments to support their children's development, with awareness of the need for language and community support, as well as support and guidance for themselves.^2^ This study reports a lack of awareness about deafness in others, negative social attitudes and a lack of access to language as the major barriers that hinder deaf people from being fully included in society.^25^ Like other studies,^26^, ^27^, ^28^ Bronfenbrenner's ecological systems theory was useful as a focus not solely on the parents and family of children who are deaf, but on the ecology into which the child is born and grows up. To study a child's development, we must look not only at the child and their immediate environment but also at the interactions of the wider environments as well.

At an individual systems level, participants spoke about attitudes, coping approaches and mostly regarding personal strength and resilience, but also the burden of feeling overwhelmed and being all‐consumed with the diagnosis of a deaf child. Determination and resilience are linked to the availability and accessibility of culturally relevant resources.^29^ Connections between protective variables and parental resilience are linked to children's quality of life.^30^

In terms of microsystems, social support is frequently highlighted as an integral part of coping experiences of parents of deaf children and often embedded within a broader framework of family‐centred service provision, with primary emphasis on perceived support from professional sources.^31^ However, for this study, many parents did not perceive support, and workers were aware of this. Similar to many public services, families and workers demonstrated awareness that support was often dependent on location, as access to services is not the same for all^32^ and access to support may be limited.^33^ More gaps in early years provision exist for deaf children, such as few staff who know British Sign Language, with recognition that it is this period before age 5 years that is vital for language acquisition.^34^

Social support is commonly categorised into four types of behaviours: emotional (hope and listening), instrumental (practical), informational (advice/suggestions) and appraisal (encouraging self‐evaluation),^35^ with all intended as beneficial. There is a relationship between received and perceived support, which also relates to well‐being. Received support is usually about the quantity of support behaviours present, while perceived support relates more to the satisfaction and availability of that support.^36^ Parents in this study were aware they received fewer services in rural areas and that this impacts on population health.^37^ However, other parents who described several support interventions reported they were only ‘muddling through’ and felt unsupported. More than one worker in this study suggested that when parents are given a diagnosis of deafness for their child, which they are not expecting, they may not take in all the suggestions and support that are on offer, implying that they do not ‘take in’ the advice, similar to other health appointments at traumatic times. Attention is pulled away from the immediate conversation to the processing of something else^38^ (e.g. the new information of diagnosis). Furthermore, there may be something here about understanding parental expectations. The expectations from services may be unrealistic and can be a source of stress, sending a message to providers that they do not fully understand the broader context of family life and limiting parent engagement instead of encouraging it.^39^

At the mesolevel, subthemes related to socialising and resource engagement, as well as others' lack of knowledge, lack of information and negative views from others. Getting together with other parents with deaf children was spoken about by many parent participants as a helpful enabler. Conversely, many hearing parents spoke about information poverty at a community level^40^ in terms of workers they connected with around their children in primary care who knew very little about deafness and parents said this made appointments with their child awkward and tense. For these relationships at the mesolevel to be more positive, such as between parents and school or primary care staff, to benefit both child and parent, there needs to be mutual trust in the way the different parties connect.^12^

The exosystem, meaning the interaction, interplay and processes that take place between two or more environments that are external to, but influence the individual^27^ included talk from participants about community and Deaf clubs, as well as multidisciplinary teams, were enablers in supporting hearing parents. The established connections between health and education professionals are a resource that could be extended to reach and support caregivers at an early stage in their children's lives by facilitating multiprofessional and parent–parent early support. Concerning barriers related to a lack of access to and knowledge about Deaf Child and Adolescent Mental Health Services,^41^, ^42^, ^43^ as well as barriers relating to bureaucracy, and a lack of understanding of wider systems. The potential of the role of deaf mentors or deaf adult role models for deaf children remains largely unknown^44^ but was mentioned by participants in this study as having the potential to support their children and in turn family members.^45^ Deaf mentors have featured in family programmes, but are not consistently available, usually due to funding issues and availability.

Considering macro issues, such as wider systems relating to policy, funding and infrastructure, one of the key areas parents spoke about was the limited access to British Sign Language classes. Whilst there is limited provision, the most pressing factor participants highlighted was the prohibitive cost of attending, as there is very limited funding available to support families to learn British Sign Language. Families are aware of the ongoing challenge of battling bureaucracy to obtain funding,^46^ although it is seldom available. The importance of access to language is vital,^47^ and like many parents in this study mentioned, signing at home is important even if their child also has the additional nurturing and care of a signing community. British Sign Language provides a host of benefits for deaf children—especially in the prevention and reduction of language deprivation. It is not just the early years of a child's life that matter for language acquisition, it is the early months, the early weeks and even the early days. Deaf children cannot wait for accessible language input.^2^ Increasing research is now focusing on the development of apps to support parents learning British Sign Language.^48^

Chronosystems, relating to human ecology structures and practices that continue to change over time, were included in participants' talk mostly with regard to a wish for clear pathways for children who are deaf through health and education services, as it was suggested this would help parents and workers to know routes and what to expect. Studies have reported how professionals with traditional training may not recognise parents' responses to deaf children and there is also a need for a greater understanding of the different models of deafness.^44^ Similarly, it is recognised that parents can experience trauma around the time of their child's deafness diagnosis,^45^ particularly around how a hearing parent is informed of his or her child's deafness, how they are helped to understand deafness, choices of communication and the emotional support they receive may influence not only the life of their child but their own and their family's health.^49^ Participants in this study also highlighted the need for early parent training, access to British Sign Language learning, deaf role models and increased social–emotional support, which is similar to other studies.^50^

**Figure 2:**
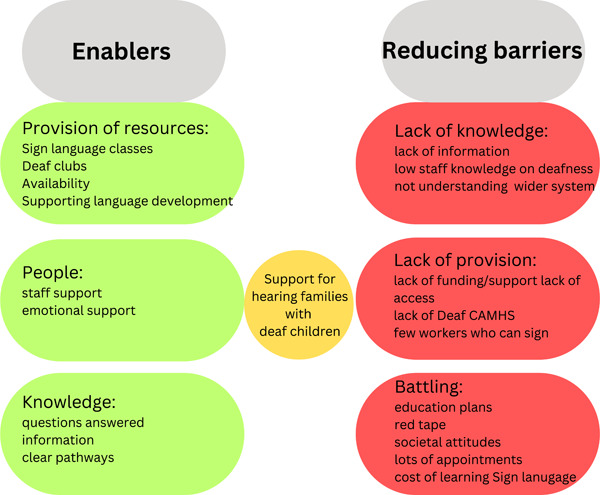
A preliminary framework of enablers and barriers in support for hearing families with children who are deaf.

A preliminary framework is proposed, which highlights the enablers and barriers that hearing parents of deaf children experience in relation to the systems and relationships in their surrounding environments (see Figure 2). The framework has been developed based on the findings from this study and is a summarised version of the overarching areas and subthemes as agreed by the steering group, and the review of published scientific literature from 2020 to 2022,^4^ and it is intended to guide future research and the development of policy and practice.

Since this study began, the Welsh Government has produced a ‘How can you become deaf aware?’ factsheet,^51^ which suggests tips on communicating with deaf children such as getting their attention first and facing them when you talk. To date, there has been a lack of evidence‐based guidance for guiding parents through complex systems about child deafness and the focus has been on information to support and encourage children, linking with education and on positive mental health, as opposed to support for parents.

As few studies currently include the voices of the parents and workers of deaf children, their contributions can help inform the challenges around navigating systems and may help those involved to respond flexibly to issues around language, education and the cognitive and social development of children who are deaf, suggesting that issues are wider than families' experiences.^49^ Factors that support hearing parents with deaf children focus on three main areas. Available resources, which include provisions to support language development, sector support (that includes emotional support) and finally knowledge, so that families have access to information and know about pathways that exist to support them and their deaf child.

### 4.2 Strengths and limitations

A strength of this study was that it included the voices of both parents and workers together. For the most part, this study was conducted during the Covid‐19 pandemic, with all data collection online, and the strength of this may have been increased accessibility and convenience for parents and workers to participate in interviews. Another strength of this study is that the findings may have relevance for hearing parents of children who are deaf in other parts of the world. By exploring the enablers and barriers, it can provide a foundation for addressing the gaps in services, policy, infrastructure and communities that stakeholders and families are keen to see improve.

Limitations of this project are that the findings are from a small country in the United Kingdom. Participants in this study were all White British, and whilst representative of the majority of communities served in Wales, they may not be generalisable to more ethnically diverse areas of the world. A further limitation was that only one researcher was involved in data collection and analysis.

## 5 CONCLUSION

In this study, participants reported that hearing parents with children who are deaf in Wales, UK experience a range of enablers and barriers during their experience of raising a deaf child. A framework of enablers and barriers in support for hearing families with deaf children has been developed from the findings of this project to demonstrate action needed for stakeholders and to inform policymakers about required change as they continue to review the accessibility of resources for deaf children and adults across age groups, provision of British Sign Language for families and what resources are required for deaf children to reach their full potential. Further engagement and provision are needed by policymakers and governments who need to ensure that language support, information and advice are prioritised across health, care and education services working with deaf children and that workers are fully educated and equipped to provide these resources. Furthermore, service providers need increased awareness that the lack of worker knowledge, limited provision and constant battling families have to engage in will surely have negative impacts on family life, stress and ultimately the health and well‐being of the deaf child. When service providers and policymakers fully recognise the support needs for hearing parents of children who are deaf, this will in turn improve the outcomes for deaf children.

## AUTHOR CONTRIBUTIONS

The sole author led on study conceptualisation, study management, data collection, analysis and writing of the manuscript. The study was part of a postdoctoral fellowship and was supervised by Professor Jaynie Rance, RCBC Wales lead at Swansea University and the project steering group.

## CONFLICT OF INTEREST STATEMENT

The author declares no conflict of interest.

## ETHICS STATEMENT

This study received ethical approval from the School of Health and Social Care, Swansea University in August 2021, and was confirmed by the Health Research Authority on 22 September 2021—21/HCRW/0024.

## Supporting information

Supporting information.

## Data Availability

The data that support the findings of this study are available on request from the corresponding author. The data are not publicly available due to privacy or ethical restrictions.
