# Supplementary material for: Enablers and barriers for hearing parents with deaf children: Experiences of parents and workers in Wales, UK
Source: Health Expect. 2023 Sep 11;26(6):2666–83. doi: 10.1111/hex.13864 (PMC10632626; doi:10.1111/hex.13864)
Supplement: Supplementary file 1 — Supporting information. [file HEX-26--s001.docx]

Interview topic guide

Study title: SUPERSTAR – systems that support hearing families with deaf children

**For Parents/workers:**

We’re here today to talk about your child who is deaf/children you work with who are deaf, and under 13 years of age.

Before we start, is there anything you would like to ask?

Please remember that we can stop at any time for a comfort break or to stop the interview entirely. I’m going to be recording this interview so that I can accurately know what you say today.

[If online interview via Zoom or equivalent – ask participant to change their online name to ‘Anon’ and tell them how to do this]

Conversational starters (adjust to parent/worker):

- North, Mid or South Wales?
- Can you tell me a bit about where you currently live/work? Is it near a big town or rural? What’s it like getting to services you use like for health or education?
- To help me understand your circumstances a bit more can you tell me if you work outside the home, and about day to day responsibilities briefly

FOR PARENTS/FAMILY MEMBERS:

- Finding out your child was deaf
  - Tell me about when you first realised your child was deaf
  - What support were you and your child offered? From which services?
  - What help was offered to other family members?
  - What things helped?
  - What were some of the challenges?
  - What about choices?
- As your child has got older, what support have you had in raising [insert child’s name]?
- From your family
- From school and education
- From the wider community and organisations
- What other support would you have liked?
- What have been the main challenges?
- What have been the barriers?
- What has helped?

FOR WORKERS:

- - Tell me about the support offered to families when they first find out their child is deaf
  - What help is usually offered to other family members?
  - What things do people say help?
  - What are some of the challenges?
  - What about choices?
- As child get older, what support do hearing parents generally have…?
- From their family
- From school and education
- From the wider community and organisations
- What other support do people say they would like?
- What have been the main challenges?
- What have been the barriers?
- What has helped?

Useful prompts:

- *Ask for examples*
- *Tell me more about…..*
- *What else would have helped?*
- *What would you like to have changed?*
- *What would you suggest to other people in a similar situation?*

Is there anything else you want add or anything that you think we haven’t covered?
